# Psychological Impacts of the COVID-19 Pandemic Among Portuguese and Swiss Higher-Education Students: Protocol for a Mixed Methods Study

**DOI:** 10.2196/28757

**Published:** 2021-06-29

**Authors:** Ana Querido, Djamel Aissaoui, Maria Dos Anjos Dixe, Françoise Schwander-Maire, Tanya Cara-Nova, Zaida Charepe, Carlos Laranjeira

**Affiliations:** 1 School of Health Sciences Polytechnic of Leiria Leiria Portugal; 2 Center for Innovative Care and Health Technology (ciTechCare) Polytechnic of Leiria Leiria Portugal; 3 Center for Health Technology and Services Research (CINTESIS) Porto Portugal; 4 School of Health Sciences Fribourg HES-SO University of Applied Sciences and Arts Western Switzerland Fribourg Switzerland; 5 Institute of Health Sciences Universidade Católica Portuguesa Lisboa Portugal; 6 Centre for Interdisciplinary Research in Health (CIIS) Universidade Católica Portuguesa Lisboa Portugal; 7 Research in Education and Community Intervention (RECI) Piaget Institute Viseu Portugal

**Keywords:** study protocol, pandemic, students, mental health, COVID-19

## Abstract

**Background:**

Higher-education students are particularly vulnerable to both everyday stressors and mental health problems. Public health emergencies may generate a range of unforeseen potential stressors for vulnerable individuals and communities. The current pandemic has apparently led to an increase in psychiatric symptoms among these students.

**Objective:**

The goal of this study is to characterize the psychological impact of the COVID-19 pandemic among Portuguese and Swiss higher-education students.

**Methods:**

This project will use a mixed methods sequential explanatory design in Portugal and Switzerland, with two consecutive phases. During Phase I, a quantitative study will assess the psychological responses of higher-education students during the COVID-19 pandemic. A convenience sampling method will be used for collecting information from students. The association between variables will be determined with univariable and multivariable analyses. During Phase II, qualitative data will be collected in order to understand the determinants of psychological stress and the strategies adopted by students as a result of the COVID-19 pandemic, as well as to identify their opinions and feelings about the teaching-learning process during quarantine. In this phase, participants will be selected using a maximum-variation sampling method. Data from focus group discussions will be coded and inductively analyzed using a thematic analysis approach. Finally, quantitative and qualitative results will be merged during interpretation to provide complementary perspectives.

**Results:**

This paper describes and discusses the protocol for this mixed methods study, which will be completed in December 2021. This study was formally approved by the local ethics committee (CE/IPLEIRIA/22/2020) in Portugal and authorized by the Swiss Association of Research Ethics Committees, swissethics (CER-VD-2020-02889).

**Conclusions:**

This research can contribute to the development of teaching tools and methods that reinforce positive mental health strategies, hope, and adaptive coping among students, and to the development of a class on mental health interventions in the context of catastrophic and traumatic events. This project will also help government stakeholders as well as health and education professionals safeguard the psychological well-being of students facing an expanding COVID-19 pandemic.

**International Registered Report Identifier (IRRID):**

PRR1-10.2196/28757

## Introduction

### Background

Disasters may have an adverse impact on the mental health of affected populations [1]. Statistics show that 20% to 40% of affected people suffer from mild changes, and 30% to 50% suffer from moderate to severe psychological distress [2,3]. Those who already had a mental disorder need more help than before, because timely mental health support reduces the chances of becoming mentally and psychologically ill [2,3]. Catastrophes are traumatic events with health implications that can affect a large number of people [4].

Events that are neither predictable nor preventable, over which people have no control, tend to have the worst consequences for people. The more serious the consequences (eg, loss of life, irreparable physical damage, and irretrievable loss of resources), the greater the need for psychosocial support [4]. People suffer from various mental health problems during and after disaster situations, so it is crucial to promote a feeling of security, tranquility, and hope and to provide access to social, physical, and emotional support [3]. Trauma-enhancing situations are opportunities to improve mental health services and develop support strategies [3].

For an adaptive response to a stressful situation, a person must develop coping strategies [5]. This biopsychosocial reaction is part of daily life. A positive reaction (ie, eustress) allows for learning and development of functional strategies, while a negative reaction (ie, distress) disrupts a person’s stability and becomes harmful [6]. Different coping strategies have been modeled in mental health research, by isolating and analyzing the behavioral, cognitive, and affective processes that subjects use during threatening events in order to control, diminish, tolerate, and/or minimize their impact on physical and psychological well-being [7]. These strategies are known as *coping strategies* and typically include coping or avoidance behaviors, management of human and material resources, social support, positive revaluation, and engagement in activities [8].

The severity a person attributes to an event will influence the development of this response [9]. When a person is unable to resolve a situation, there is a maladaptive response, which increases vulnerability to both physical and psychological diseases [5]. Some pre-existing mental illnesses can get worse after a disaster, such as depression, alcoholism, or schizophrenia. Other conditions can be induced by situations such as bereavement: alcohol or drug abuse, anxiety, and posttraumatic stress disorders. However, not all people who experience such situations will need support; most will recover favorably over time, finding a way to resume their routines [3]. Statistics indicate that in the coming decades, the number of disasters could increase and affect human development indicators, such as life span, health, education, and standard of living [10]. It is, therefore, necessary to provide and improve access to long-term mental health services. Professionals should be trained on how to intervene in disaster situations and acquire strategies to deal with difficulties and to prevent disease [2]. Nursing interventions integrated into daily routines are relevant to rebuilding-life projects, promoting feelings of hope, and addressing more complex situations. Greater awareness of the mental health issues caused by disasters is increasingly relevant [11].

The ongoing COVID-19 pandemic, caused by SARS-CoV-2, is promoting physical and physiological health problems, including fear, anxiety, and high stress levels. The development of appropriate mental health assessments and treatments is urgently needed [12]. Previous research revealed a profound and wide range of psychosocial impacts during infectious disease outbreaks at the individual, community, and international levels. On an individual level, people are likely to experience fear of becoming sick or dying themselves, feelings of helplessness, as well as stigma [13].

At the time of writing (May 2, 2021), more than 150 million people around the world had been infected and more than 3.1 million had died from COVID-19 [14]. In Switzerland, from January 3, 2020, to May 2, 2021, there were 667,557 confirmed cases of COVID-19 and 10,057 deaths. A total of 2.8 million vaccine doses were administered. In Portugal during the same period, there were 838,475 confirmed cases of COVID-19 and 16,988 deaths. A total of 3.4 million vaccine doses were administered [15]. The differences between both countries spring from a combination of factors, such as personal values, social and cultural background, gender roles, and education levels [16]. Moreover, the degree of impact was also affected by the level of national fiscal health before the COVID-19 crisis. While Switzerland has experienced strong fiscal health and possessed a well-equipped health care system to face the crisis, Portugal and its National Health Service have experienced, since 2010, economic austerity and health care budget cuts, whose effects were exacerbated during this pandemic [17].

Portugal and Switzerland faced the pandemic differently, and distinct societal and economic characteristics between the two countries can have impacts on the mental health of each population. Currently, there is very little available information on the psychological impact and mental health of the general public during the peak of the COVID-19 pandemic. Based on our preliminary analysis of the literature, much of the research related to this pandemic focuses on the epidemiology and the clinical characteristics of infected patients [18], the genomic characterization of the virus, the challenges for global health governance [19], and economic impacts [20]. In contrast, there seems to be a scarcity of research articles examining the psychosocial impact of COVID-19 on the general population or specific groups (elderly, children, students, etc).

### Psychological Impact of the COVID-19 Pandemic Among Higher-Education Students

The *student* stage constitutes an important transition in an individual’s life. Certain recurring elements concerning mental health and well-being can be observed during this period, such as an increase in anxiety and stress, the appearance of depressive states, or a high risk of exhaustion. In some recent studies, such problems have been found in up to 50% of higher-education students [21-23]. Numerous studies have used coping strategies as an explanatory model regarding mental health problems among students (eg, stress, anxiety, and psychological distress) [24-26].

While these epidemiological data are important, most were collected prior to the COVID-19 pandemic. During 2020, most national governments implemented sanitary health measures to stop or slow down the transmission of the virus responsible for the COVID-19 pandemic. Numerous measures were imposed on the population, such as closing schools and public places, instituting widespread quarantine, requiring social distancing, and almost completely ceasing recreational and cultural activities.

The response to the COVID-19 health emergency also disrupted academic and social life in many universities, increasing anxiety and distress among students [23]. Many universities cancelled their activities and closed campuses, shifting to online classes or ending their semesters early, leading many students to return home. Given the travel restrictions, many international students were not able to return to their home countries. Students from underprivileged families, with marginal housing or other financial or physical challenges, faced disproportionate amounts of difficulty finding last-minute transportation and housing. Furthermore, at home, some students lacked the technological infrastructure to follow classes online and engage with their, now virtual, social community. Students in quarantine risked losing opportunities critical to their scholarly or professional advancement [27,28]. These circumstances could understandably further social isolation and perpetuate stress, anxiety, and low mood. Even students not under quarantine and from well-resourced families faced stress due to the sudden changes and uncertain climate of relocations, changes in the academic schedule, and the shift to online teaching [28,29]. The combination of regular academic stress, compounded by the academic changes in response to the pandemic, and the general experience of a potentially traumatic public health crisis may lead to clinically significant psychiatric symptoms and illness among higher-education students [30].

Moreover, this population, mostly between the ages of 18 and 25 years, is a particularly at-risk population [31], open to very significant consequences, both psychological (ie, mental health) and social (ie, educational attainment). However, not all students suffer from psychological difficulties. Some seem to have resources and coping strategies that enable them to diminish or even counter the current situation’s adverse effects. Therefore, it is necessary to identify these strategies and evaluate them, in order to propose possible solutions and improvements when caring for students, and to provide them with assistance and high-level education.

One meta-analysis of 27 studies from 15 countries, analyzing the psychological impact of COVID-19 among college students, showed a disproportionate burden of mental health problems among participants, with females having higher anxiety and depression levels than males. Increased stress, anxiety, and depressive symptoms were seen as a result of changes and uncertainty in university education, technological concerns about online courses, being far from home, social isolation, decreased family income, and future employment [32]. These impacts were also observed in universities across the world [33-36].

A similar scenario was found in Portugal. One study observed high levels of anxiety, depression, and stress among a population of 460 university students [37]. In Switzerland, a study by Elmer et al [38] revealed that students’ levels of stress, anxiety, loneliness, and depressive symptoms got worse with the pandemic, compared to previous levels. Stressors shifted from fears of missing out on social life to worries about health, family, friends, and their future. The results suggest that concerns regarding COVID-19, lack of interaction and emotional support, and physical isolation were associated with negative mental health trajectories.

We currently have little data on student reactions to the COVID-19 pandemic, from both national studies and international comparisons. The latter could be important to compare the solutions instituted in different countries and to suggest possible solutions. All these elements support the implementation of a policy for assessing student mental health with a standardized methodology and, if possible, an international scope, since policy choices are more or less similar.

### Project Description

This study will investigate the psychological impact of the COVID-19 pandemic among higher-education students in Portugal and Switzerland. In this research, we are interested in the following: (1) assessing the prevalence of psychological symptoms, (2) correlating risk and protective factors related to psychological stress with student characteristics, (3) comparing the levels of psychological symptoms between both countries, (4) identifying strategies adopted by students to promote better psychological adjustment during the pandemic crisis, and (5) characterizing the perception and experience of the teaching-learning process during quarantine.

Our research questions are as follows: (1) How has the COVID-19 pandemic affected student mental health? (2) What individual strategies were adopted to deal with the consequences of the COVID-19 pandemic? and (3) What challenges did students face when adapting to the teaching-learning process during the pandemic?

A quantitative approach can reveal how risk factors (ie, depression, stress, and anxiety) and protective factors (ie, hope, resilience, and coping) are correlated with student characteristics, such as level of education, social support (ie, living alone or in a family context), knowledge and perception of the illness, and perception of the effectiveness of protective measures against COVID-19. 

In addition, levels of stress and anxiety are expected to be correlated with coping strategies, hope levels, and the perception of the impact of events on oneself and one’s surroundings. 

A qualitative approach can help us understand the adjustment patterns and the impact of the COVID-19 pandemic on student work and potential psychological consequences. Furthermore, we also intend to clarify student perceptions about new teaching-learning processes compared to in-person teaching. 

## Methods

### Type of Study

In order to carry out our research, we chose a cross-sectional study with a mixed methods sequential explanatory design (Multimedia Appendix 1). This approach consists of two distinct phases: a quantitative phase followed by a qualitative phase [39]. In this design, a researcher first collects and analyses the quantitative (ie, numeric) data, and then collects and analyses the qualitative (ie, text) data. The latter can help explain or elaborate on the quantitative results obtained in the first phase. In this approach, the quantitative data and their subsequent analysis provide a general understanding of the research problem [40], while the qualitative data and their analysis refine and explain those statistical results by exploring participant views in greater depth [39,40].

During Phase I, we will carry out a cross-sectional survey, in the Portuguese and Swiss contexts, to assess the psychological responses of higher-education students during the COVID-19 pandemic using an anonymous online questionnaire.

In Phase II, a qualitative approach will be used to understand the psychological stress experienced by students due to the COVID-19 pandemic, focusing on their perceptions, attitudes, and experiences. Information will be collected from online focus group discussions (FGDs). According to Liamputtong [41], this methodology is appropriate for sensitive topics and vulnerable populations. The FGDs will be useful for exploring differences of opinion and will provide an opportunity to delve deeper into the phenomena under study through an interactive discussion of participant experiences and attitudes, in a group context.

### Recruitment and Sampling

Data will be collected in two stages. All potential participants will be given written information on the study and asked to provide their informed consent. Participants will include adults at any level of education beyond high school, including undergraduate and graduate programs. Students participating in Erasmus or other mobility programs will be excluded.

First, we will collect data using an online survey created by the researchers specifically for this purpose, including its items, protocol, and variables. We will contact the deans of each institution to present our research and obtain authorization to disseminate our online survey to students. A convenience sampling method will be used to collect information from potential participants. We will use mailing lists to send individual emails containing our questionnaire, an explanation of our research, and the results of our research. The assumption of nonduplication of response will be taken into account by limiting one response per email account.

For Portugal, sampling will take place in four higher-education institutions (ie, universities and polytechnic institutes). Given a total population of 40,000 students—an average of 10,000 students per institution—our sample size should include 381 students, for a confidence level of 95%.

For Switzerland, sampling will occur within the HES-SO (Haute école spécialisée de Suisse occidentale) University of Applied Sciences and Arts of Western Switzerland, covering the cantons of Vaud; Neuchâtel, a French-speaking region; Fribourg; and Valais, where French is the co-official language. According to a recent survey, the total student population was approximately 21,000 in the 2019-2020 academic year [42]. The size of our sample should, therefore, include 377 students, for a confidence level of 95%.

After the online surveys, we plan to carry out at least two online FGDs in each context (ie, Portuguese and Swiss). Participants who complete the anonymous online survey will have the option to provide their contact information (eg, phone number or email). The participants representing diverse backgrounds, in terms of gender, education, and geography, will be selected using a maximum-variation sampling method. Eligible students will be contacted by email, and we will send them the documentation concerning the informed consent and the interview guides. If they are interested in participating in the FGDs, we will schedule appointments with the student groups. The FGDs will take place using the Microsoft Teams videoconferencing platform in groups of 6 to 10 participants, with facilitation by the researchers. This will give a total sample of 24 to 40 participants. The sample size should provide sufficient data to meet our aims and cover a range of views. Data collection and analysis will continue until thematic saturation is reached in the inductive analysis of qualitative interviews. A key advantage of the Microsoft Teams platform is its ability to securely record and store sessions without recourse to third-party software [43].

### Data Collection and Instruments

#### Phase I: Survey

The e-questionnaire constructed by our project team will be based on FAQ (frequently asked questions) found on the World Health Organization official website [44].

Our questionnaire will include questions related to eight items. The first series of questions, pertaining to items 1 to 4, will have to be developed for, and linguistically adapted to, both countries. Linguistic discrepancies that might arise when ensuring cross-cultural congruency or equivalency between the two languages will be solved by a bilingual research team member. The second series of questions pertain to items 5 to 8. The eight items are as follows:

1. Sociodemographic and health information, including variables such as gender; age; geographical region; education; marital status; employment, if applicable; household size; perceived health status; and history of chronic disease.
2. Contact history, including close contact with a person with confirmed COVID-19 infection, indirect contact with a person with confirmed COVID-19 infection, and contact with a person with suspected COVID-19 infection or infected materials.
3. Knowledge and perceptions of COVID-19, including (1) knowledge of transmission routes, level of satisfaction with COVID-19 health information, trends in new cases and deaths, and potential treatment of COVID-19 infection; (2) sources of information; and (3) concerns about COVID-19 variables, such as COVID-19 infection of self and other family members and chances of survival if infected.
4. Protective measures against COVID-19, including (1) avoiding the use of shared utensils during meals, conduct in case of coughing and sneezing, and hygiene rules and (2) individual and social measures to avoid COVID-19.
5. Mental health status, which will be measured using the 21-item Depression, Anxiety, and Stress Scale (DASS-21) developed by Lovibond and Lovibond [45]. The total depression subscale score was divided into scores for normal depression (0-9), mild depression (10-12), moderate depression (13-20), severe depression (21-27), and extremely severe depression (28-42). The total anxiety subscale score was divided into five score categories: normal (0-6), mild anxiety (7-9), moderate anxiety (10-14), severe anxiety (15-19), and extremely severe anxiety (20-42). The total stress subscale score was divided into scores for normal stress (0-10), mild stress (11-18), moderate stress (19-26), severe stress (27-34), and extremely severe stress (35-42). The DASS-21 has been shown to be a reliable and valid measure for assessing the mental health of the Portuguese-speaking [46] and French-speaking [47] populations.
6. The psychological impact of COVID-19, which will be measured using the Impact of Event Scale–Revised (IES-R) developed by Weiss and Marmar [48]. The IES-R is a self-administered questionnaire that has been validated in the Portuguese population to determine the extent of psychological impact after exposure to a public health crisis [49]. A French version of the scale is also available [50]. This 22-item questionnaire is composed of three subscales and aims to measure average avoidance, intrusiveness, and hyperarousal. The total IES-R score was divided into the following score categories: normal (0-23), mild psychological impact (24-32), moderate psychological impact (33-36), and severe psychological impact (>37) [51].
7. Coping strategies, which will be measured using the Brief Resilient Coping Scale developed by Sinclair and Wallston [52]. This is a 4-point measure designed to assess an individual’s tendency to cope adaptively. The cutoff point categories are low resilience (4-13), medium resilience (14-16), and high resilience (17-20). The Portuguese version was validated by Pais-Ribeiro and Morais [53], and the French version was translated by Ionescu [54].
8. Hope levels, which will be assessed using the Herth Hope Index (HHI) created by Herth [55]. Hope has been recognized in all disciplines as an important motivational state for overcoming adversity or life-threatening situations. The HHI measures different dimensions of hope using a 4-point Likert scale, ranging from 1 (strongly disagree) to 4 (strongly agree). The scale has an overall score ranging from 12 to 48, as well as individual scores ranging from 1 to 4. A higher score indicates a higher level of hope. The Portuguese version was validated by Viana et al [56]. No translation or cross-cultural evaluation of the HHI into French has yet been published. Therefore, we will conduct a methodological validity study of the instrument following the COSMIN (Consensus-Based Standards for the Selection of Health Measurement Instruments) guidelines [57] and by using a reverse method for transcultural validation [57]. A pilot study will be carried out prior to data collection and will include two phases: Phase I will comprise the adaptation of the scale, including translation and back translation, an expert evaluation, and a pretest [57], and Phase II will involve validation of the translated scale, including assessment of reliability and factorial validity of all the scale’s items. To establish the initial psychometric properties of the HHI, at least 120 higher-education students will be recruited. According to the general rule of thumb, the sample size should be at least 10 individuals per item for an exploratory factor analysis or principal component analysis [58].

#### Phase II: Focus Group Questions

Findings from the online survey will serve as a basis to develop questions to facilitate discussion in the focus groups, as demonstrated in Multimedia Appendix 1. A qualitative description provides a straightforward, rich account of experiences to understand the perspective of individuals, as well as the rationale for their actions [39].

Two key considerations in cross-national focus group research is that questions asked in each country cover the same themes and be understood similarly by participants in each country [59]. We will define a fixed number of broad questions to be discussed for 20 to 30 minutes each. An initial 5-minute icebreaking round of introductions will force every participant to say something early on, preventing people from remaining silent throughout the discussion. Furthermore, moderators in both countries will be instructed to simply refer participants back to the question asked, limiting follow-up questions to the clarification of opinions. In each country, the same nondirective moderating style will be followed to promote interaction between focus group participants. Standardizing the moderating style will also improve the cross-national comparability of the discussions.

Interviews will be structured to identify participant perceptions and attitudes about the determinants of psychological stress, strategies adopted by students to promote better psychological adjustment during the pandemic crisis, and students’ opinions and feelings about the teaching-learning process during quarantine. At this point and based on the current state of evidence [21,60,61], certain external and environmental variables have been identified as relating to mental health, as follows: (1) new forms of academic work organization (eg, distance education and evaluation), (2) personal working conditions and study strategies, and (3) perceived social isolation and interactions with others (eg, classmates and educational staff).

### Data Analysis

#### Quantitative Measures

Quantitative data from the standardized measures will be entered into SPSS Statistics, version 27 (IBM Corp), for analysis. Parametric and nonparametric techniques—as appropriate, depending on the nature and distribution of the data—will be used to estimate and explain changes in primary and secondary outcomes. Descriptive data analysis will be performed using absolute and relative frequencies, mean and standard deviation, and median and interquartile range. The appropriate techniques will analyze the association and/or differences between groups according to the conditions (chi-square tests, *t* tests, analyses of variance, etc). Associations between variables will be determined with univariable and multivariable analyses, using simple linear regression and general linear regression analyses, respectively. The significance level will be set at α=.05.

#### Qualitative Measures

The interviews will be audio-recorded and transcribed in full, with all personal and place identifiers removed. Data will be managed and analyzed with the aid of the computer-assisted qualitative data analysis software package ATLAS.ti (Scientific Software Development GmbH). The data from FGDs will be coded and inductively analyzed using a thematic analysis approach [62]. Inductive codes will be created and used to identify and link segments of data in a variety of meaningful ways. Verbatim quotes from participants, translated from the original Portuguese and French versions into English, will be used as examples when presenting results. Categories will be represented visually using diagrams to illustrate the conceptual relationship between the emerging categories. Data analysis will be guided by the COREQ (Consolidated Criteria for Reporting Qualitative Research) guidelines [63].

## Results

This study is currently ongoing and the results, using the recruitment, data collection, and data analysis methodology described above, are expected to be available no later than the end of 2021.

### Triangulation of Quantitative and Qualitative Data Findings

According to the sequential explanatory design discussed in Multimedia Appendix 1, an initial data analysis will be undertaken when completing Phase I (ie, quantitative data extracted from e-questionnaires). The results of Phase I will help develop an interview guide for the FGDs with students. In Phase II, qualitative data will be analyzed using thematic analysis, and the results of this phase will be integrated with the previous analysis to obtain a broader and more accurate picture describing psychological needs during the COVID-19 pandemic among higher-education students. In this study, both quantitative and qualitative cultural data sets will be collected independently and researchers in each study will integrate data sets into four stages: (1) data collection, by designing qualitative interview questions based on the results of the quantitative survey instrument; (2) sampling, by selecting interviewees for the qualitative phase from among participants in the quantitative phase; (3) coding process (ie, hierarchical tree); and (4) inferential stage, by jointly discussing the results from both quantitative and qualitative approaches and comparing the corresponding analysis maps generated in the study.

As noted previously, ensuring the conceptual equivalence of constructs, the functional and linguistic equivalence of instruments, and the authentic comparability of translated data are critically important issues for all cross-cultural research [64]. The presence of a bilingual researcher on our team constitutes a linguistic advantage to actively intervene in data integration, by systematically establishing lexical equivalence across languages in the online survey (ie, quantitative) and capturing authentic oral meanings and articulating them in transcriptions (ie, qualitative).

The criteria of trustworthiness and authenticity will be taken into account [65]. Trustworthiness will be provided by elements such as the study’s duration and the use of triangulation of sources, methods, and researchers. To ensure study reliability, the researchers will be deeply involved in data handling and will maintain transparency while they analyze the data. The research team includes six nurses involved in academic teaching and with expertise in nursing practice (AQ, CL, MD, FSM, TC, and ZC), as well as a psychologist (DA).

Qualitative data from both countries will be shared, and procedural similarities and differences in each context will be discussed. Field and self-reflective notes will be taken during data collection and analysis to enhance transparency and provide an audit trail of context and on how key decisions on interpretation were made [64]. For that purpose, we will use qualitative coding techniques (ie, interrater reliability) to ensure study trustworthiness, because multiple researchers will be involved with coding. Multiple competing realities and perspectives may differ across time and context, and our findings will be limited to the time and context of this study. Transferability of findings is nonetheless maximized by triangulation to ensure the inclusion of different perspectives. Findings will be used to produce recommendations and best practices.

### Ethical Considerations

Ethical rules and considerations will follow the international Declaration of Helsinki guidelines [66]. Requests for ethical approval differ between the two countries participating in this research. For Portugal, this study was formally approved by the local ethics committee (CE/IPLEIRIA/22/2020). For Switzerland, ethics approval was given by the Swiss Association of Research Ethics Committees, swissethics (CER-VD-2020-02889).

Personal data will be processed in accordance with the European Union General Data Protection Regulation (GDPR2016/679) and, until the end of the study, only the research manager will have access to data identifying the participants. All participants will be informed of the purpose of the study, that participation is voluntary, that they can leave the study at any time, and that all data are handled confidentially. By responding to and submitting the e-survey, participants will give their consent to participate in the study. Similarly, an independent informed consent form will be presented to the students participating in the focus groups, stating the same principles and reinforcing the anonymization and confidentiality of the information shared during the FGDs.

Quantitative data processing and analysis will be carried out by the research team, and the use of files will be the responsibility of the two main researchers. Data transfer will respect the Swiss federal law on data protection, as well as the law of the canton of Fribourg [67] and regulation No. 2016/679, known as the General Data Protection Regulation for Portugal. A contract between the two parties supports these elements.

## Discussion

### Study Rationale

As with any other type of natural disaster, the risk of a pandemic cannot be eliminated. Despite prevention efforts, pandemics will continue to occur and will, at times, overwhelm the systems in place to mitigate health, societal, and economic effects [68]. As stated in Madhav et al, “Unlike most other natural disasters, pandemics do not remain geographically contained, and damage can be mitigated significantly through prompt intervention. As a result, there are strong ethical and global health imperatives for building capacity to detect and respond to pandemic threats, particularly in countries with weak preparedness and high spark and spread risk” [68]. There is no single, optimal response to a public health emergency; strategies must be tailored to the local context and to the severity and type of pandemic [68].

Considering the relevance of global warming and the increase in frequency and magnitude of natural disasters, mental health assessments should be extended to impacts caused by the pandemic. There is little understanding of mental well-being assessment under pandemic scenarios involving social isolation and lockdown. This study will enable us to conduct a multisite survey in Portugal and Switzerland on the perception of the psychosocial impact of the COVID-19 pandemic among higher-education students, using the same research instruments. By studying and comparing the current situation in both countries, this study could provide empirical evidence for psychiatric symptoms and coping strategies. Thus, the results could inform the development of psychosocial interventions to minimize psychological impact, anxiety, depression, and stress due to the COVID-19 pandemic [69] and provide a baseline for evaluating prevention and control efforts for future biological catastrophes.

This mixed methods international study will also contribute to the development of pedagogical strategies and good practices to reinforce positive mental health strategies, hope, and adaptive coping during catastrophes and traumatic events. These elements would enable institutions to be recognized as mental health friendly.

### Conclusions

In any biological disaster, themes of fear, uncertainty, and stigmatization are recurrent and may act as barriers to appropriate medical and mental health interventions [12]. Based on experience from past, serious, novel pneumonia global outbreaks and the psychosocial impact of viral epidemics, the development and implementation of mental health assessment, support, and services are crucial and pressing goals for a health care response to the COVID-19 pandemic [69]. The recognition that the academic community’s recovery after a disaster will need to include resilience and mental health promotion can do much to improve our understanding of mental health and its determinants more broadly. We expect that our findings will promote other multicenter studies in other contexts, based on our Portuguese and Swiss partnership.

## Appendix

Multimedia Appendix 1 Summary of the sequential explanatory mixed methods research design.

## Abbreviations

COREQ: Consolidated Criteria for Reporting Qualitative Research
COSMIN: Consensus-Based Standards for the Selection of Health Measurement Instruments
DASS-21: 21-item Depression, Anxiety, and Stress Scale
FAQ: frequently asked questions
FGD: focus group discussion
HES-SO: Haute école spécialisée de Suisse occidentale
HHI: Herth Hope Index
IES-R: Impact of Event Scale–Revised
