# Supplementary material for: Psychological Impacts of the COVID-19 Pandemic Among Portuguese and Swiss Higher-Education Students: Protocol for a Mixed Methods Study
Source: JMIR Res Protoc. 2021 Jun 29;10(6):e28757. doi: 10.2196/28757 (PMC8244726; doi:10.2196/28757)
Supplement: Multimedia Appendix 1 [file resprot_v10i6e28757_app1.docx]

Multimedia Appendix 1. Summary of the sequential explanatory mixed methods research design.

- Online Focus Groups

- Maximum variation sampling method

- Thematic analysis approach

- Online Survey

- Convenience sampling method

- Uni and multivariable analysis

**Triangulation**

**Quantitative Research**

**Qualitative Research**

**Integration of the Quantitative and Qualitative results**

1

2

3

3
